# Home-Based Treatment with Immunoglobulins: an Evaluation from the Perspective of Patients and Healthcare Professionals

**DOI:** 10.1007/s10875-018-0566-z

**Published:** 2018-11-12

**Authors:** Chantal A. Zuizewind, Paul van Kessel, Christine M. Kramer, Mary M. Muijs, Janneke C. Zwiers, Mattanja Triemstra

**Affiliations:** 1 NIVEL, Netherlands Institute for Health Services Research, Otterstraat 118-124, 3513 CR Utrecht, The Netherlands; 2 Sanquin Plasma Products BV (SPP), Plesmanlaan 125, 1066 CX Amsterdam, The Netherlands

**Keywords:** Immunoglobulins, home infusion therapy, evaluation study, treatment outcome, shared decision-making, quality of life

## Abstract

**Purpose:**

This study aims to evaluate home-based treatment with immunoglobulin (IgG) by assessing and comparing the experiences and perceived value of patients and healthcare professionals, and potential differences in experiences between subcutaneous (SCIg) and intravenous (IVIg) modes of administration. As choices on the location and type of treatment are determined in a shared decision-making process, we evaluated the home-based treatment from the perspectives of both patients and professionals.

**Methods:**

A questionnaire study was conducted among 205 patients, 44 informal caregivers, 43 hospital professionals, and 21 nurses of the Sanquin Home Service (SHS) that provides home treatment with immunoglobulins in the Netherlands. Experiences, perceived benefits, and effects on the patients’ quality of life and overall ratings were assessed.

**Results:**

Both patients and professionals were predominantly positive about the home treatment, irrespective of the administration mode. The home-based treatment with Ig contributed to the patients’ autonomy, participation, and perceived health. Patients and informal caregivers valued the treatment with a global rating of 8.84, and professionals with 8.32 (on a scale from 0 “worst” to 10 “best possible care”). SCIg and IVIg patient groups differed in their experiences regarding the accessibility and communication of the home treatment service. Furthermore, hospital professionals reported lower effects on quality of life than patients themselves.

**Conclusions:**

Home-based treatment with immunoglobulins is highly valued because of its personalized and effective character, meeting the needs and preferences of patients. Nonetheless, patients and professionals do have different perspectives on the value of this type of care.

**Electronic supplementary material:**

The online version of this article (10.1007/s10875-018-0566-z) contains supplementary material, which is available to authorized users.

## Introduction

Immunoglobulin replacement therapy is a common treatment for patients affected with various immunologic deficiency syndromes. It aims to increase the serum immunoglobulin G (IgG) level to normal physiologic concentrations and to protect against bacterial infections [1]. In addition, Ig therapy is used for immune modulation in several auto-immune and neurological conditions. Ig therapy can be provided in a hospital or, if well tolerated, at home. The administration mode is either intravenously (IVIg) or subcutaneously (SCIg). Both modes of administering IgG have been demonstrated to provide adequate protection from infections and are well tolerated [2]. Each mode has its strengths and weaknesses, and the choice of administration mode depends on a shared decision-making process where physician’s judgment, patient’s preferences, medical or individual needs, and sometimes the insurance coverage are taken into account [3–5].

Treatment with Ig has a positive effect on the health-related quality of life (HRQoL) of patients with immunologic deficiency syndromes [1, 6]. Nevertheless, the treatment scheme is often intensive and burdensome and may be required chronically. The Ig must constantly be replenished at specific intervals, depending on individual needs and mode of administration. Whereas IVIg is generally administered in relatively large volumes once every 3–4 weeks, SCIg is given in smaller more frequent doses, for example, once or twice a week. Patients switching from IVIg replacement therapy in the hospital or doctor’s office to home-based SCIg administration showed strong improvements in HRQoL [7–11]. They not only felt more energetic, but they also reported increased emotional and social well-being and less restrictions on daily activities such as work and school. Furthermore, the introduction of home-based Ig therapy has shown to increase treatment satisfaction. Home treatment with SCIg is evaluated as more convenient, comfortable, and flexible and leads to greater independence in patients than hospital-based treatment with IVIg [9, 12].

In most countries, home-based Ig treatment is given solely subcutaneously. In Europe, aside from the UK, Sweden, France, and the Netherlands [13, 14], home-based intravenous infusion with Ig is still uncommon. Consequently, previous research on home-based Ig therapy predominantly focused on subcutaneous home treatment, often compared to intravenous treatment in hospitals. Literature on the effects of intravenous home treatment is scarce, and the few available studies are dated or concerned small numbers of patients with specific indications [15–17]. Hence, a complete and thorough evaluation of user experiences and effects of home-based therapy for both administration modes is lacking. Moreover, previous studies on home-based Ig treatment have not taken into account both the patients’ and healthcare professionals’ perspectives. As the choice of location and mode of administration is based on shared decision-making of patients and healthcare professionals, an in-depth evaluation of the home treatment from different perspectives is of great importance. Therefore, the aim of this study is to evaluate home-based Ig treatment by assessing and comparing user experiences and the perceived value of home treatment for both patients and healthcare professionals, and to study potential differences between subcutaneous and intravenous modes of administration.

This study presents an evaluation of home treatment with IgG in the Netherlands. Although the majority of patients with immunologic deficiency syndromes is treated within a hospital setting (i.e., standard care), there is a rising number of patients in the Netherlands switching to home therapy after first receiving IgG treatment in the hospital. Currently, there are five service providers for home therapy with IgG in the Netherlands, and the Sanquin Home Service (SHS) is one of them. SHS operates independently and the service is not limited to specific plasma product brands. This service aims to provide personalized care at home to patients who receive immunoglobulin therapy by offering a variety of different schemes for home-based treatment, including subcutaneous and intravenous treatment and ranging from home-based infusion of Ig by a qualified nurse to guidance of patients and informal caregivers in the self-administration. Patients are referred to home-based treatment by the physician in the hospital, after the patient, informal caregiver(s), and physician together have decided that this treatment option is safe and appropriate given the medical and individual needs of the patients. The hospital-based physician remains responsible for the patient at all time and is kept informed by means of a web-based reporting system. In general, patients with SCIg treatment are trained by SHS nurses to self-administer and continue treatment by themselves after approximately three supported sessions. Patients with IVIg treatment receive permanent assistance in administering IgG, either from a nurse or an informal caregiver. Though the venipuncture is generally performed by the nurse, some patients are trained to execute specific steps in the procedure (e.g., removing the needle afterwards, programming the infusion pump). In all cases, SHS nurses are available as backup when needed.

This study offers an exceptional opportunity to compare both administration modes (SCIg and IVIg) in the home setting with the regular treatment (IVIg) in hospitals, and to evaluate the home treatment from the perspectives of all persons involved (i.e., patients, informal caregivers, and healthcare professionals).

## Methods

### Study Design and Population

Patients or their informal caregivers (i.e., parents, spouses) and healthcare professionals of the SHS filled in an online survey. All patients who recently or previously have been treated with immunoglobulins by the home service provider (in 2014–2015, or in the case of (ex) pregnant women, in the past 5 years) were approached for participation. Also, hospital professionals who prescribe home-based treatment to patients (specialists and specialized nurses) and the professionals involved in performing the treatment at home (qualified nurses) were asked to participate.

Patients and informal caregivers (*n* = 656) were invited to participate in the study by the pharmacy (Mediq) and healthcare professionals by SHS. They were contacted by letter with a request to register for the study through an online or written registration form. After registration, they received a personal e-mail with a link to an online questionnaire. Healthcare professionals (*n* = 156) were invited per e-mail by SHS to participate in the survey. Potential respondents received two reminders for completing the questionnaire, 2 and 4 weeks after the first request. Informed consent was obtained from all individual participants included in the study.

### Questionnaires

Two questionnaires were developed (in Dutch): one for patients and their informal caregivers, and another for healthcare professionals [18]. Draft versions of the questionnaires were based on focus group discussions and interviews with patients, informal caregivers, and healthcare professionals. In addition, we used literature and other relevant questionnaires as example. The questionnaires were pre-tested in 21 cognitive interviews with 11 patients and 10 professionals to evaluate the clarity and content validity of the questions [19], and the questionnaires were adjusted accordingly (see Appendices 1 and 2 for the final questionnaires (translated into English)).

The questionnaire for patients and informal caregivers consisted of 155 questions and two overall ratings concerning the home-based service and hospital care (i.e., global rating and recommendation score). The questionnaire for healthcare professionals consisted of 90 questions and overall ratings. An overview of all constructed scales, from both questionnaires, can be found in Appendix 3 (see also “Statistical Analysis”).

The global ratings concerning the home-based service and hospital care (addressed in both questionnaires) had answering scales from 0 (worst possible care) to 10 (best possible care). Recommendation scores assessed the probability of recommending the service to others (0 = not at all; 10 = very likely) [20]. And the fear of needles was measured with an 11-point scale ranging from 0 (no fear at all) to 10 (extremely anxious). All other questions were positively formulated propositions, with a 5-point answering scale: (− 2) completely disagree, (− 1) partly disagree, (0) agree nor disagree, (1) partly agree, (2) completely agree. We chose a default of positively formulated questions, as this best resembled the experiences reported in the focus groups and interviews on which the questionnaires were based.

### Statistical Analysis

Data were analyzed by using Stata, version 14.1. First, psychometric analysis was conducted to assess the dimensional structure of the questionnaires (see Appendix 3 for the 20 constructed scales, their underlying items, and internal consistency (Cronbach’s alpha)). Secondly, scores were calculated for each questionnaire and response group, either based on separate items or on reliable scales (alpha ≥ 0.70). Two scales for the comparison of the perspectives of clients and professionals showed moderate reliability (accessibility and communication: alpha = 0.61; perceived benefits of SHS: alpha = 0.58), but were included for descriptive purposes.

Differences in experiences and overall ratings between patients, informal caregivers, and the two types of healthcare professionals were examined with analysis of variance and consecutive post hoc tests. In addition, potential differences between patients with various administration modes (SC or IV) were examined by analysis of variance with correction for differences in the patients’ gender and age. A *p* value of < 0.05 was used as limit for statistical significance.

To compare the perspective of clients and healthcare professionals, similar items from the two questionnaires were used to compute comparable scale scores (see Appendix 3 (bottom part) for the five scales regarding the various perspectives on experiences, perceived benefits, and effects of home treatment). In the comparative analysis, the two types of healthcare professionals (SHS nurses and hospital professionals) are regarded as separate groups.

## Results

### Response and Background Characteristics of Respondents

#### Response

A total of 656 patients or informal caregivers (together called “clients”) were invited to participate in the study, of which 286 indicated their interest in participation by registering through an online or written registration form. A number of 249 filled out the questionnaire, resulting in a response percentage of 38% of the invited client group and 87% of those who registered for the study. Of the 249 client respondents, 205 were patients of SHS and 44 were informal caregivers. A total of 156 professionals (125 hospital professionals and 31 SHS nurses) were invited to participate in the study, of which 64 (43 hospital professionals and 21 nurses) filled out the questionnaire. This resulted in a response percentage of 41%.

A comparison between the invited client group (*n* = 656) and the respondents (*n* = 249) showed that those who filled out the questionnaire were on average about 3 years older than the whole target population. There were no significant differences in gender, administration mode, and indication.

#### Description of Respondents

Table 1 summarizes the demographics, clinical, and other background characteristics of the respondents. Patients had a mean age of 51.9 years, and most of them were women (61%), treated for an immunological disorder (69%) with intravenous administration (79%). Patients for which an informal caregiver filled out the questionnaire were younger (mean age 33.1 years), mainly affected with an immune disorder (89%), and treated intravenously (55%). Altogether, the main indication for IgG treatment was an immune disorder (72%; mostly PID and SID, some auto-immune disorders), followed by neurological conditions (25%), pregnancy or gynecological problems (2%), or another indication like Crohn’s disease (1%).

**Table 1:** Demographics and background characteristics of respondents (clients and professionals)

	Clients		Healthcare professionals	
	Patients (*n* = 205)	Informal caregivers^a^ (*n* = 44)	SHS nurses (*n* = 21)	Hospital professionals (*n* = 43)
Gender				
Male (%)	78 (38%)	28 (64%)	6 (29%)	19 (44%)
Female (%)	119 (58%)	14 (32%)	15 (71%)	23 (54%)
Unknown	8 (4%)	2 (4%)		1 (2%)
Age				
Mean (sd)	51.9 (14.2)	33.1 (25.3)	51.1 (8)	46.3 (11)
Indication				
Immune disorder (%)	141 (69%)	39 (89%)	–	–
Neurological (%)	57 (28%)	4 (9%)	–	–
Pregnancy (%)	6 (2.5%)	0	–	–
Other/unknown (%)	1 (0.5%)	1 (2%)	–	–
Administration mode				
Intravenous	161 (79%)	24 (55%)	–	–
Subcutaneous	44 (22%)	20 (46%)	–	–
Person administering the medication				
SHS nurse	149 (73%)	22 (50%)	–	–
Patient	35 (17%)	0 (0%)	–	–
Informal caregiver	9 (4%)	18 (41%)	–	–
Other	12 (6%)	4 (9%)	–	–
Duration of treatment				
< 6 months	34 (17%)	1 (2%)	–	–
6–12 months	26 (13%)	9 (20%)	–	–
1–2 years	37 (18%)	9 (20%)	–	–
3–5 years	61 (30%)	13 (30%)	–	–
6–10 years	46 (22%)	12 (27%)	–	–
Changes in treatment procedure^b^				
Change in administration mode (from IVIg to SCIg or vice versa)	24 (12%)	6 (14%)		
Temporary switch to hospital	11 (5%)	3 (7%)	–	–
Permanent switch to hospital	2 (1%)	1 (2%)	–	–
Profession				
SHS nurse	–	–	21 (100%)	
Hospital doctor/specialist	–	–		32 (74%)
Hospital nurse	–	–		11 (26%)
Working experience				
Less than 1 year	–	–	0	5 (12%)
1 to 3 years	–	–	3 (14%)	7 (16%)
3 to 6 years	–	–	7 (33%)	12 (28%)
6 to 10 years	–	–	11 (52%)	19 (44%)

^a^Characteristics refer to the patients for whom a questionnaire was filled out

^b^Multiple answers possible

Of the patient respondents, 73% of the infusions were performed by a SHS nurse and 17% by the patients themselves (and 10% by another person). With regard to the patients for which an informal caregiver filled out the questionnaire, 50% of the infusions were done by a SHS nurse and 50% by the informal caregiver or another person (e.g., other parent or relative). At the time of the study, no patients received facilitated subcutaneous immunoglobulin (fSCIg) nor rapid push SCIg.

Most patients received home treatment for at least 3 years (53–57%). A minority of patients changed the administration mode (12–14%) over the course of their IgG therapy. Switches from IVIg to SCIg are generally resulting from a wish to become more independent or to reduce side effects, whereas switches from SCIg to IVIg are mostly to initiate by the need to administer higher volumes or a wish to reduce the treatment frequency. Also, some patients temporarily (5–7%) or permanently (1–2%) switched back to hospital-based treatment, because of a change in the clinical condition or side effects to therapy that require a hospital setting.

The participating SHS nurses were on average 51.1 years, mainly women (71%) with more than 6 years of experience working for SHS (52%). The hospital professionals had a mean age of 46.3 years and 44% of them worked more than 6 years for SHS.

### Clients’ Perspective

#### Experiences

**Fig. 1:**
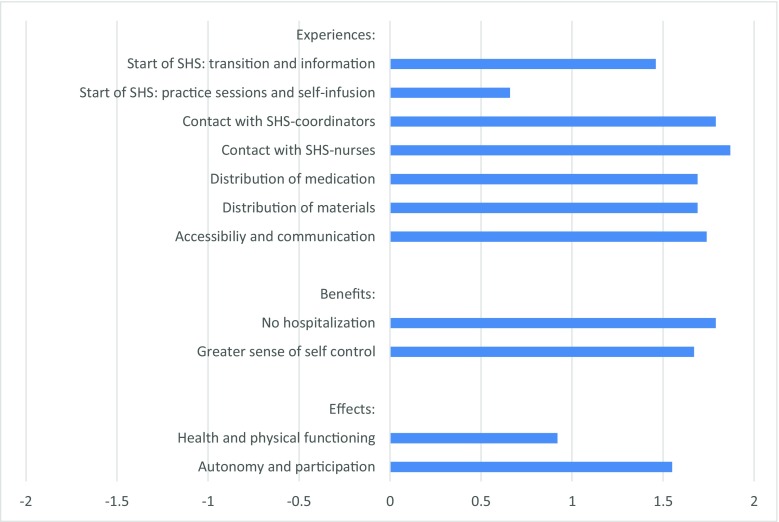
Experiences, perceived benefits, and effects of SHS as reported by patients (*n* = 205) and informal caregivers (*n* = 44). Questions formulated as propositions, with a 5-point answering scale: − 2, completely disagree; − 1, partly disagree; 0, agree nor disagree; 1, partly agree; 2, completely agree. Bars represent mean scores

Patients and their informal caregivers predominantly reported positive experiences with the home-based service (see Fig. 1). The respondents were most positive about their contacts with the coordinators and nurses of the SHS, and somewhat less positive about the start of the home treatment. The experiences differed only slightly between patients and informal caregivers and between patients with different modes of administration. In general, the informal caregivers showed the most positive experiences and they reported a better start of the home treatment than patients (mean = 1.67 and 1.41 respectively; *p* < 0.05), being more positive about the transition from the hospital to the home setting and the information provided. Patients receiving Ig through subcutaneous infusion (mean = 1.86) were more positive about the accessibility and communication of the home-based service than clients receiving Ig intravenously (mean = 1.70; *p* < 0.05).

#### Fear of Needles

**Fig. 2:**
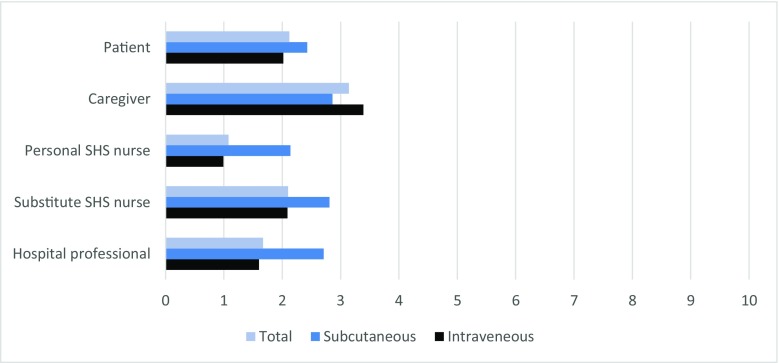
Fear of needles reported by patients with intravenous (*n* = 161) or subcutaneous (*n* = 44) administration modes. Answering scale: 0, no fear at all; 10, worst possible fear. Bars represent mean scores

Figure 2 illustrates patients’ fear of needles, taking into account the administration mode and different treatment procedures. Patients with intravenous Ig treatment reported lower fear of needles than patients with subcutaneous Ig treatment. This was both the case for their experiences with treatment by nurses in the hospital (mean = 1.60 and 2.71 respectively; *p* < 0.05) and for the treatment by their personal SHS nurse at home (mean = 0.99 and 2.14 respectively; *p* < 0.001). Overall, patients’ fear of needles was the lowest when injected by their personal SHS nurse.

#### Perceived Benefits

Patients and their informal caregivers experienced several benefits of home treatment over hospital treatment. The most frequently mentioned benefits of the home-based service concerned less hospitalization and an increased sense of self-control. Patients and informal caregivers reported the advantages of treatment taking place in the own environment, not having to travel to the hospital, not having to be in the hospital, and less chance of (hospital acquired) infections. Furthermore, patients and informal caregivers reported advantages of having the possibility to determine day and time of administration, to adjust the rate of infusion to their own preferences (e.g., more prompt if the patient is occupied, or slower if that reduces side effects), and to control and administer the Ig product at a comfortable temperature (e.g., sufficient time to warm the product to room temperature). The reported benefits did not differ between patients and informal caregivers, nor between patients receiving intravenous or subcutaneous Ig administration.

#### Effects on Quality of Life

Patients and informal caregivers experienced two main effects of the home treatment on their lives: effects on their *perceived health and physical functioning*, and effects on their *autonomy and participation in daily life* (see lowest part of Fig. 1). There were no significant differences in the perceived effects of home treatment between patients and informal caregivers, nor between patients receiving intravenous or subcutaneous Ig administration.

#### Overall Ratings

Clients evaluated the home-based service with an average global rating of 8.84 (sd = 1.15) on a scale from 0 “very poor care” to 10 “excellent care.” This appraisal was nearly one point higher than their global rating of the previously received hospital-based treatment, which was on average 7.85 (sd = 1.65). The positive appraisal of home treatment was also reflected in the willingness of patients and informal caregivers to recommend home treatment with Ig to others. Almost all patients and informal caregivers (99%) seemed to recommend the home-based service, as they gave a recommendation score of 6 or higher (on a scale from 0 “definitely not recommended” to 10 “definitely recommended”), and 55% of them gave a maximum score of 10. Once again, no differences were found in appraisal and recommendation of the home-based service between patients and informal caregivers and patients with different administration modes.

### Professionals’ Perspective

#### Experiences

**Fig. 3:**
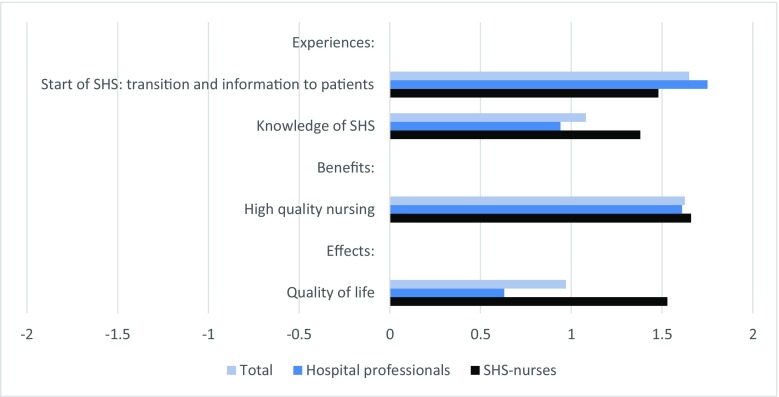
Experiences, perceived benefits, and effects of SHS according to hospital professionals (*n* = 43) and SHS nurses (*n* = 21). Questions formulated in propositions, with a 5-point answering scale: − 2, completely disagree; − 1, partly disagree; 0, agree nor disagree; 1, partly agree; 2, completely agree. Bars represent mean scores

Figure 3 presents the mean scores of the healthcare professionals on constructed scales with regard to the start of the home treatment (transition and information to patients) and the knowledge of professionals regarding the SHS. Hospital professionals were more positive about the transition patients go through when switching from hospital to home treatment and the complementary information provision than the SHS nurses (mean = 1.75 vs. 1.48; *p* < 0.05). On the other hand, SHS nurses reported to have more knowledge of SHS than the hospital professionals did (mean = 1.38 vs. 0.94; *p* < 0.05).

#### Perceived Benefits

Healthcare professionals also reported their perceptions of the benefits of home treatment for patients. One major benefit stood out, and that is the quality of SHS nurses (Fig. 3). Nearly all professionals acknowledged the advantage of the use of a small and permanent group of specialized nurses, who know the patient personally and have thorough knowledge of the home treatment. Other benefits mentioned by nearly all professionals were the fact that treatment takes place in the patients’ own environment, patients not having to travel, and time benefits for patients. Different perspectives were observed regarding two benefits. SHS nurses perceived a reduced risk of (hospital acquired) infections and the possibility of adjusting home treatment (e.g., infusion rate) to the patients’ preferences more strongly as benefits of home treatment than hospital professionals did (respectively 100% vs. 79% completely agree, *p* < 0.01; and 95% vs. 76%, *p* < 0.05).

#### Effects on Quality of Life

The perceived effect of home treatment on the patients’ quality of life differed between professionals. SHS nurses were more convinced of a positive effect than the hospital professionals (mean = 1.53 vs. 0.63; *p* < 0.0001).

#### Overall Ratings

Healthcare professionals evaluated the home treatment with an average global rating of 8.32 (sd = 0.78), ranging between 6 and 10 (on a scale from 0 “very poor care” to 10 “excellent care”). There was no significant difference in the appraisal by hospital professionals and SHS nurses. In accordance with the positive overall rating, healthcare professionals were also very willing to recommend the home treatment with Ig to others. Ninety-three percent of all healthcare professionals gave a recommendation score of 8 or higher, and 22% of the healthcare professionals gave the maximum score of 10. SHS nurses had a significant higher recommendation score than hospital professionals (mean *=* 9.15 vs. 8.54; *p* < 0.05).

### Comparison of Perspectives on SHS

Table 2 illustrates the perspectives of clients, SHS nurses, and hospital professionals with regard to their global rating of SHS and willingness to recommend home treatment to others in need of comparable care. As shown, clients appeared to be most satisfied. Their global rating for the SHS was the highest of all groups and significantly differed from the global rating of hospital professionals (mean difference = 0.61; *p* < 0.01). Furthermore, clients and hospital professionals differed significantly with regard to their willingness to recommend SHS to others, with patients being more willing to recommend SHS than hospital professionals (mean difference = 0.64, *p* < 0.01).

**Table 2:** Comparisons of the perspectives of patients, informal caregivers, SHS nurses, and hospital professionals regarding their overall ratings of SHS, perceived benefits, and effects of SHS

	Clients mean (sd)	SHS nurses mean (sd)	Hospital professionals mean (sd)	*F* test *p* value
Global rating (0 to 10)	8.84 (1.15)	8.50 (0.83)	8.23 (0.74)	*p* < 0.01
Recommendation score (0 to 10)	9.18 (1.25)	9.15 (0.81)	8.54 (0.88)	*p* < 0.01

**Fig. 4:**
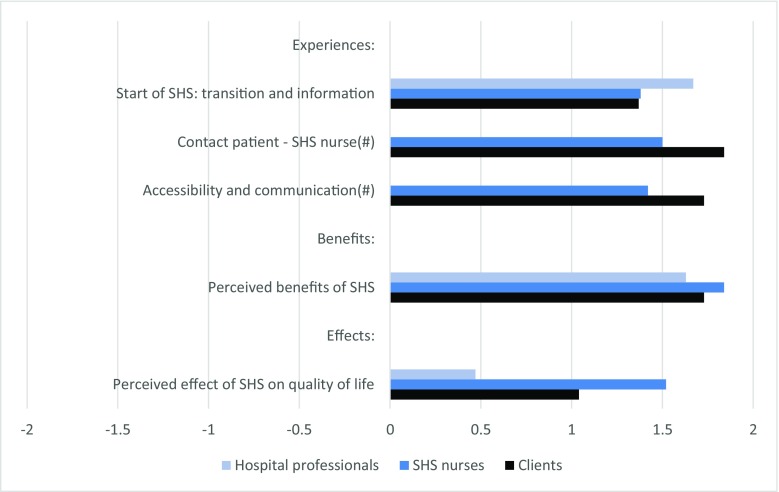
Comparison of perspective of clients (*n* = 249), SHS nurses (*n* = 21), and hospital professionals (*n* = 43) regarding their experiences, perceived benefits, and perceived effect of SHS home treatment on quality of life. Scores based on a selection of similar items, rated by both clients and healthcare professionals (see Appendix 1). Questions formulated in propositions, with a 5-point answering scale: (− 2) completely disagree, (− 1) partly disagree, (0) agree nor disagree, (1) partly agree, (2) completely agree. Bars represent mean scores. Number sign indicates scores only apply to clients and SHS nurses (no scores available for hospital professionals)

Figure 4 illustrates the perspectives of the three response groups (clients, SHS nurses, and hospital professionals) for comparable measures of experiences, perceived benefits, and effects on the patients’ quality of life. Clients were more positive than SHS nurses about their mutual contacts and the accessibility of the home-based service. Similar experiences were reported by the three groups regarding the start of the SHS and the perceived benefits. One distinction was found for the perceived effect on quality of life, with hospital professionals (mean = 0.47) reporting a significantly lower effect (*p* < 0.05) than clients and SHS nurses (1.03 and 1.52 respectively; *p* < 0.05).

## Discussion

This study assessed the patients’ and professionals’ perspective on the home treatment with subcutaneous or intravenous immunoglobulin replacement therapy by the Sanquin Home Service. The positive results of this evaluation suggest that the home treatment of SHS is highly personalized, effective, and well adapted to the needs and preferences of the target group. Both patients and professionals are predominantly positive about the SHS and highly appreciate the home-based treatment. Clients value the home treatment with a global rating of 8.84 on a scale from 0 to 10, nearly one point higher than their appraisal of the hospital-based treatment with Ig (7.85). Healthcare professionals value the SHS with an overall rating of 8.32.

The treatment at home enables and encourages the autonomy and participation of patients in daily life and contributes to their health. This applies both to subcutaneous and intravenous modes of home treatment, as nearly no differences were found in experiences and ratings of patients with these two administration modes. However, patients by whom the Ig is administered subcutaneously are more positive about the accessibility and communication of the SHS staff (nurses and coordinators) than patients receiving the Ig intravenously. As this latter patient group is more dependent on the SHS nurses in receiving their Ig product, it is likely that they are more critical towards the accessibility and communication with the SHS staff because these aspects are especially important to them.

Furthermore, patients and informal caregivers reported less positive experiences with the start of the SHS, regarding the practice sessions and the administration of Ig, than professionals did. It is likely that unfamiliarity with the procedures at the start of the home-based treatment explains this result. Similarly, unfamiliarity also seems to play a role in the fear of needles, as this appears to be higher when injected by a substitute or random nurse at home or in the hospital, and this fear was the lowest when injected by the usual and well-known SHS nurse (see Fig. 2). It can be assumed that the location of treatment may have a certain impact as patients are more likely to feel comfortable in their own environment and this may influence their perceived level of anxiety. In addition, the administration mode also seems to be related to the fear of needles, as patients receiving IVIg reported less fear than patients receiving SCIg. Because of the quasi-experimental and non-randomized design of this study, we cannot offer certainty about cause or effect; a fear of needles may very well affect the choice of administration mode, but also could be a consequence of it.

Finally, this study revealed a discrepancy between the perspectives of clients and hospital professionals, showing that the professionals gave a lower global evaluation of the home-based service and rated the effects of the service on the quality of life lower than patients.

### Strengths and Limitations of This Study

The study provides insight into the practice of home treatment with immunoglobulin, being one of the first to evaluate both subcutaneous as intravenous home treatment and taking into account the different perspectives of both patients and professionals. The results presented are in line with a substantial number of earlier findings displaying the positive evaluation of home-based treatment with SCIg and/or IVIg [7–11, 15, 16]. Nonetheless, a number of possible limitations should be considered when interpreting the results of this study.

Firstly, this study assesses home-based treatment with immunoglobulin of solely one provider (out of five) in the Netherlands, which may raise questions concerning the objectivity of the professional respondents. However, these professionals are not employed by SHS, as the home service relies on a national network of authorized and independent nurses from (local) home care organizations, and the hospital-based professionals work together with but are not in service of the SHS. Furthermore, the SHS nurses are not personally known to the physicians in the hospitals, and the nurses were not asked to rate their own performance.

Secondly, this study solely included clients who currently or recently received treatment by the SHS, and has no comparator group. Although this study also enrolled patients who recently stopped the home-based treatment, or who switched back to hospital treatment, we were not able to further analyze the experiences of these particular respondents due to the small number (*n* = 17).

In addition, the response to both questionnaires was only moderately high; 38% of the clients and 41% of the professionals responded to the survey. Though these percentages are common in healthcare evaluation surveys, it raises questions regarding the representativeness of this study. Nevertheless, non-response analyses showed no major differences between the characteristics of the client group invited to participate and the final respondents. Unfortunately, nothing is known about the representativeness of the professional respondents, as background characteristics of the non-responding professionals were not available. The relatively low response rate of the hospital professionals, with only about one third of the invited professionals who actually responded (vs. 69% of the SHS nurses), possibly reflects a lower level of involvement of hospital professionals with the SHS. However, as it is unclear how many of the professionals actually received and opened the e-mail with the invitation to fill out the questionnaire, this response rate is rather a lower bound.

### Recommendations

Home treatment seems suitable to and is almost equally preferred by patients receiving SCIg or IVIg therapy. However, the differences found in this study regarding the experiences with SCIg and IVIg home-based therapy illustrate the importance of keeping a close focus on the treatment procedures, which are different for the two administration modes. It is advisable to keep this distinction salient in future research to further optimize home-based treatment for both patient groups. Also, we recommend further research into the needs and experiences of the different patient groups who receive home-based treatment with IgG. Because the neurologic and immunological indications are likely to result in different needs, with some conditions requiring only temporary IgG therapy while others may deteriorate in due course, the HRQoL and needs regarding home treatment may be impacted differently. Moreover, instead of asking patients to compare their views on treatment at home with their (prior) treatment in hospital, it may be more informative to compare the responses of two groups of patients: those who currently receive home treatment and those who continue to receive or who switched back to treatment in hospital. Future research among both patient groups (outbound and home treatment) should thus reveal actual differences in patient experiences in the two settings.

Switching to home treatment will expose patients to a certain degree of unfamiliarity and newness. Results of this study show that this is particularly true for the experiences with practice sessions and the administration of Ig. With proper guidance at the start of the home treatment and sufficient practice sessions of administration, the Ig product might accelerate the transition to a point where clients feel accustomed and at ease with home-based treatment. To ensure a smooth transfer to home-based treatment, the use of a hospital-based transfer team, with dedicated nurses who are familiar with IgG treatment and the organizations providing home treatment, could be helpful.

This study focused at different perspectives of all persons involved, as the decision on whether to receive home- or hospital-based Ig therapy is ultimately based on the shared decision-making of patients, informal caregivers, and healthcare professionals. Because of the discrepancy found between the patients’ and professionals’ perspectives, and given the key role of the hospital professionals in providing reliable information to (potential) patients, it is recommended to better inform these professionals about the actual effects of home-based treatment on the patients’ quality of life. Thus, enabling them to provide more reliable patient information, in order to optimize the process of shared decision-making regarding the location and administration mode of Ig therapy.

## Conclusions

Home-based treatment by SHS with SCIg or IVIg is a highly valued therapy for various patient groups. The time has come to act accordingly and implement home-based treatment with Ig on a global scale to enable patients to choose a treatment option that best fits their medical condition, individual needs, and personal preferences. This research contributes to the transparency in choosing a treatment option when facing Ig treatment and facilitates patients, informal caregivers, and their healthcare professionals in the shared decision-making process concerning the location and type of treatment.

## Electronic Supplementary Material

ESM 1 (DOCX 148 kb)

ESM 2 (DOCX 108 kb)

ESM 3 (DOCX 25 kb)

## Data Availability

The data analyzed during the current study are available from the corresponding author on reasonable request.
