# Supplementary material for: Home-Based Treatment with Immunoglobulins: an Evaluation from the Perspective of Patients and Healthcare Professionals
Source: J Clin Immunol. 2018 Nov 12;38(8):876–85. doi: 10.1007/s10875-018-0566-z (PMC6292972; doi:10.1007/s10875-018-0566-z)
Supplement: Supplementary file 1 — (DOCX 148 kb) [file 10875_2018_566_MOESM1_ESM.docx]

**
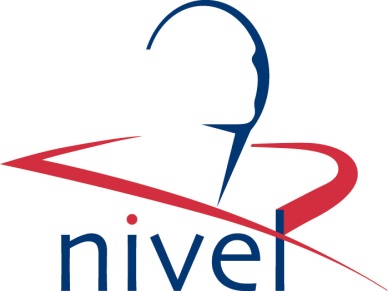
Appendix 1.**


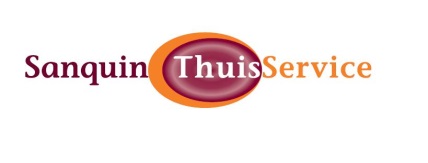


Questionnaire

**Sanquin Home Service**

Experiences of patients

**Note:**

This is a translation of a Dutch digital questionnaire for the patients or informal caregivers. The original questionnaire is included as an appendix in the Dutch research report (Triemstra et al., 2015) which can be downloaded from the Nivel-website (www.nivel.nl).

**General questions**

*You receive or have received medication through the Sanquin Home Service. Please answer the following questions regarding your personal situation.*

1. **Do you currently receive Sanquin Home Service?**
   - Yes
   - No, not anymore, but I did receive it in the past 12 months
   - No, not anymore, but I did receive it more than 12 months ago
   - No, I never used the services of the Sanquin Home Service
2. **For what purpose do you use the Sanquin Home Service?**
   - A neurological condition
   - An immunological condition
   - Due to pregnancy
   - Other: …………………………………………………………………
3. **Who uses the Sanquin Home Service?**
   - Me
   - My child
   - My partner
   - Someone else (for whom I fill in the questionnaire)

***Please note****: Do you fill in the questionnaire for another person? Below we refer to 'you' as the patient (or your child, spouse or other person), unless we ask for your own opinion or experience as a parent or informal caregiver.*

1. **How long have you used the Sanquin Home Service?**
   - Less than half a year
   - 6 to 12 months
   - 1 to 2 years
   - 3 to 5 years
   - 6 to 10 years
2. **Did anything change to your home treatment during this period? *(multiple answers possible)***
   - Other medication
   - Other dosage of the medication
   - Other administration mode (from intravenous to subcutaneous or vice versa)
   - Temporarily ceased the home treatment
   - Ceased the home treatment and referred back to treatment at the hospital
   - Completely ceased treatment (no treatment anymore)
   - Other: ………………………………………………………………….
   - No changes

***Please note:*** *If the home treatment has changed, please complete the questionnaire for the current or most recent treatment mode as delivered to you by the Sanquin Home Service.*

1. **Who administers the medication from the Sanquin Home Service?**
   - Nurse of the Sanquin Home Service
   - Patient him-/herself
   - Spouse
   - Parent
   - Someone else: ………………………………………………………………….
2. **How is the medication from the Sanquin Home Service administered?**

- Intravenous (infusion in a blood vessel)
- Subcutaneous (infusion under the skin)

1. **Which product (medication) do you receive from the Sanquin Home Service?**

Intravenous:

- - Nanogam
  - Gammagard
  - Kiovig
  - Flebogamma dif.
  - Other: ………………………………………………………………….
  - I don’t know

Subcutaneous:

- - GammaQuin
  - Subcuvia
  - HyQvia
  - Other: ………………………………………………………………….
  - I don’t know

1. **How often is the medication from Sanquin Home Service administered to you?**
   - Every day (daily)
   - 2 to 3 times a week
   - Once a week (weekly)
   - Once every two weeks (biweekly)
   - Once every three weeks
   - Once every four weeks or monthly
   - Other: ………………………………………………………………….
2. **How was the medication previously administered in the hospital?**
   - Intravenous (infusion in a blood vessel or port-a-cath)
   - Subcutaneous (under the skin)
   - Both (a period) intravenous and (a period) subcutaneous
   - Not applicable (I received no medication at the hospital)
3. **Do you have experience with different modes of administration at home?**
   - Yes, both intravenously and subcutaneously
   - No, only intravenously
   - No, only subcutaneously

**Start of the Sanquin Home Service***The following questions concern the start of the home treatment and the transition of treatment in the hospital to treatment at home.*

*Please answer the questions by indicating to what extent you agree with the statements. If you have no experience regarding a particular subject, please answer ‘not applicable’.*

|  | ***Strongly disagree*** | ***Partially disagree*** | ***Neither agree, nor disagree*** | ***Partially agree*** | ***Strongly agree*** | ***Not applicable*** |
| --- | --- | --- | --- | --- | --- | --- |
| 1. **The transition from hospital treatment to home treatment went well.** |  |  |  |  |  |  |
| 1. **Beforehand, I knew what to expect from the home treatment.** |  |  |  |  |  |  |
|  | | | | | | |
|  | ***Strongly disagree*** | ***Partially disagree*** | ***Neither agree, nor disagree*** | ***Partially agree*** | ***Strongly agree*** | ***Not applicable*** |
| 1. **Beforehand, I have received sufficient information regarding…** | | | | | |  |
| 1. **…the processes of the Sanquin Home Service.** |  |  |  |  |  |  |
| 1. **…the delivery of medication and materials at home.** |  |  |  |  |  |  |
| 1. **…the home treatment’s costs.** |  |  |  |  |  |  |
|  | | | | | | |
|  | ***Strongly disagree*** | ***Partially disagree*** | ***Neither agree, nor disagree*** | ***Partially agree*** | ***Strongly agree*** | ***Not applicable*** |
| 1. **I received sufficient information about the Sanquin Home Service at the start of the treatment from…** | | | | | |  |
| 1. **…the physician in the hospital** |  |  |  |  |  |  |
| 1. **…the nurse in the hospital** |  |  |  |  |  |  |
| 1. **…the coordinators of the Sanquin Home Service** |  |  |  |  |  |  |
| 1. **…the nurse of the Sanquin Home Service** |  |  |  |  |  |  |

|  | | ***Strongly disagree*** | ***Partially disagree*** | ***Neither agree, nor disagree*** | | ***Partially agree*** | | ***Strongly agree*** | ***Not applicable*** |
| --- | --- | --- | --- | --- | --- | --- | --- | --- | --- |
| 1. **Looking back, I would have liked to receive information about the possibility of home treatment earlier.** |  | |  |  |  | |  | |  |
| 1. **I wish the Sanquin Home Service would have started earlier.** |  | |  |  |  | |  | |  |

*Please answer the following questions regarding the guidance of the nurses of the Sanquin Home Service in administering the medication.*

1. **Did you receive training from a nurse before administering the medication independently (by yourself)?**
   - No, not applicable (medication is still administered by the nurse) *[-> question 23]*
   - Yes, 1 or 2 practice sessions
   - Yes, 3 or 4 practice sessions
   - Yes, 5 or more practice sessions

*Please indicate to what extent you agree with the statements. If you have no experience regarding a particular subject, please answer ‘not applicable’.*

|  | ***Strongly disagree*** | ***Partially disagree*** | ***Neither agree, nor disagree*** | ***Partially agree*** | ***Strongly agree*** | ***Not applicable*** |
| --- | --- | --- | --- | --- | --- | --- |
| 1. **The number of practice sessions in administering the medication was sufficient** |  |  |  |  |  |  |
| 1. **After the final practice session, I felt confident to infuse myself.** |  |  |  |  |  |  |
| 1. **During the starting period, I was uncomfortable administering the medication myself.** |  |  |  |  |  |  |
| 1. **During the starting period, I thought the self-infusion was scary.** |  |  |  |  |  |  |

1. **What could be improved regarding the start of the Sanquin Home Service?**
   - Nothing
   - The following:

|  |
| --- |

**Contact with the coordinators of the Sanquin Home Service**

*The following questions are about your contact with the coordinators of the Sanquin Home Service.*

*Please answer the questions by indicating to what extent you agree with the statements. If you have no experience regarding a particular subject or if you don’t know what to answer, please choose ‘I don’t know / not applicable’.*

| 1. **The coordinator of the Sanquin Home Service…** | ***Strongly disagree*** | ***Partially disagree*** | ***Neither agree, nor disagree*** | ***Partially agree*** | ***Strongly agree*** | ***I don’t know / Not applicable*** |
| --- | --- | --- | --- | --- | --- | --- |
| 1. **…takes sufficient time for me** |  |  |  |  |  |  |
| 1. **…is aware of my situation** |  |  |  |  |  |  |
| 1. **…listens to me carefully** |  |  |  |  |  |  |
| 1. **…takes me seriously** |  |  |  |  |  |  |
| 1. **…takes my wishes and needs into account** |  |  |  |  |  |  |
| 1. **…answers my questions well** |  |  |  |  |  |  |
|  | | | | | | |
| 1. **I have faith in the expertise of the coordinator** |  |  |  |  |  |  |
| 1. **I feel emotionally supported by the coordinator** |  |  |  |  |  |  |

1. **What could be improved regarding your contact with the coordinator of the Sanquin Home service?**
   - Nothing
   - The following:

|  |
| --- |

**Contact with the nurses of the Sanquin Home Service**

*The following questions are about your contact with the nurses of the Sanquin Home Service who come to your home for administering the medication.*

1. **How many different nurses of the Sanquin Home Service have visited you at home during the past year for administering the medication?** *If no nurses visited your home, please answer ‘0’.*

… nurses *[if 0 -> question 38]*

1. **How do you feel about the number of nurses that have visited you at home for administering the medication?**
   - Too few
   - Just right
   - Too much
   - Way too much
   - No opinion
2. **Do you have a regular (personal) nurse from the Sanquin Home Service?**
   - Yes
   - No *[-> question 32]*
   - I don’t know *[-> question 32]*
3. **I get along well with my personal nurse (there’s a perfect match)**
   - Strongly disagree
   - Partially disagree
   - Neither agree, nor disagree
   - Partially agree
   - Strongly agree
   - I don’t know

*Please answer the questions by indicating to which extent you agree with the statements. If you don’t know the answer to the question, please answer ‘I don’t know’.*

| 1. **The (personal) nurse of the Sanquin Home Service…** | ***Strongly disagree*** | ***Partially disagreed*** | ***Neither agree, nor disagree*** | ***Partially agree*** | ***Strongly agree*** | ***I don’t know*** |
| --- | --- | --- | --- | --- | --- | --- |
| 1. **…takes sufficient time for me** |  |  |  |  |  |  |
| 1. **…is aware of my situation** |  |  |  |  |  |  |
| 1. **…listens to me carefully** |  |  |  |  |  |  |
| 1. **…takes me seriously** |  |  |  |  |  |  |
| 1. **…takes my wishes and needs into account** |  |  |  |  |  |  |
| 1. **…keeps appointments** |  |  |  |  |  |  |
| 1. **…is competent in administering injections.** |  |  |  |  |  |  |
| 1. **…works hygienically** |  |  |  |  |  |  |
| 1. **…could answer all my questions about the home treatment** |  |  |  |  |  |  |

*Please answer the following questions by indicating to which extent you agree with the statements. If you have no experience regarding a particular subject, or if you don’t know what to answer, please choose ‘I don’t know’/ not applicable.*

|  | ***Strongly disagree*** | ***Partially disagree*** | ***Neither agree, nor disagree*** | ***Partially agree*** | ***Strongly agree*** | ***I don’t know / Not applicable*** | |
| --- | --- | --- | --- | --- | --- | --- | --- |
| 1. **I have faith in the expertise of the nurse.** |  |  |  |  |  |  | |
| 1. **I feel emotionally supported by the nurse .** |  |  |  |  |  |  | |
| 1. **The nurses of the Sanquin Home Service collaborate well.** |  |  |  |  |  |  | |
|  | | | | | | | |
| 1. **The substitute nurses…** | ***Strongly disagree*** | ***Partially disagree*** | ***Neither agree, nor disagree*** | ***Partially agree*** | ***Strongly agree*** | ***I don’t know / Not applicable*** |  |
| 1. **…are well informed regarding my situation** |  |  |  |  |  |  |  |
| 1. **…are competent in administering the injections** |  |  |  |  |  |  |  |

1. **What could be improved regarding your contact with the nurses of the Sanquin Home Service?**
   - Nothing
   - The following:

|  |
| --- |

**Distribution of medication**

*The following questions concern the delivery of the medication (immunoglobulins) of the Sanquin Home Service.*

*Please answer the questions by indicating to which extent you agree with the statements. If you have no experience regarding a particular subject, answer with ‘Not applicable’.*

|  | ***Strongly disagree*** | ***Partially disagree*** | ***Neither agree, nor disagree*** | ***Partially agree*** | ***Strongly agree*** | ***Not applicable*** |
| --- | --- | --- | --- | --- | --- | --- |
| 1. **The delivery of medication runs smoothly** |  |  |  |  |  |  |
| 1. **I always know exactly what type and dosage of medication will be delivered.** |  |  |  |  |  |  |
| 1. **Scheduling appointments for the delivery of medication runs smoothly.** |  |  |  |  |  |  |
| 1. **I am well informed about the time of delivery.** |  |  |  |  |  |  |
| 1. **Medication is delivered in the agreed time period.** |  |  |  |  |  |  |
| 1. **I am informed on time if the medication cannot be delivered in the agreed time period.** |  |  |  |  |  |  |
| 1. **Medication is delivered on time.** |  |  |  |  |  |  |
| 1. **Dosages are always delivered correctly.** |  |  |  |  |  |  |
| 1. **I know what to do in case of problems with the medication delivery.** |  |  |  |  |  |  |

1. **What could be improved regarding the delivery of the medication?**
   - Nothing
   - The following:

|  |
| --- |

**Distribution of materials**

*The following questions concern the delivery of materials (such as infusion pump, needles, bandage materials) for the Sanquin Home Service.*

*Please answer the questions by indicating to which extent you agree with the statements. If you have no experience regarding a particular subject, answer with ‘Not applicable’*

|  | ***Strongly disagree*** | ***Partially disagree*** | ***Neither agree, nor disagree*** | ***Partially agree*** | ***Strongly agree*** | ***Not applicable*** |
| --- | --- | --- | --- | --- | --- | --- |
| 1. **The delivery of materials runs smoothly.** |  |  |  |  |  |  |
| 1. **Scheduling appointments for the delivery of materials runs smoothly.** |  |  |  |  |  |  |
| 1. **Materials are delivered on time.** |  |  |  |  |  |  |
| 1. **I am well informed about the time of delivery.** |  |  |  |  |  |  |
| 1. **Materials are delivered in the agreed time period.** |  |  |  |  |  |  |
| 1. **I am informed on time if materials cannot be delivered in the agreed time period.** |  |  |  |  |  |  |
| 1. **I have enough space to store all of the materials at home.** |  |  |  |  |  |  |
| 1. **I know what to do in case of problems with the delivery of materials.** |  |  |  |  |  |  |

1. **What could be improved regarding the delivery of the materials?**
   - Nothing
   - The following:

|  |
| --- |

**Accessibility and communication**

*The following questions concern the accessibility by telephone and communication of the employees of the Sanquin Home Service (coordinators and nurses).*

*Please answer the questions by indicating to which extent you agree with the statements. If you have no experience regarding a particular subject, please answer ‘Not applicable’.*

|  | ***Strongly disagree*** | ***Partially disagree*** | ***Neither agree, nor disagree*** | ***Partially agree*** | ***Strongly agree*** | ***Not applicable*** |
| --- | --- | --- | --- | --- | --- | --- |
| 1. **The SHS coordinators are available when needed.** |  |  |  |  |  |  |
| 1. **Telephone contact with the coordinators is running smoothly.** |  |  |  |  |  |  |
| 1. **The nurse of the Sanquin Home Service is easily accessible.** |  |  |  |  |  |  |
| 1. **Telephone contact with the SHS nurse is running smoothly.** |  |  |  |  |  |  |
| 1. **I get adequate support when there are problems with the medication.** |  |  |  |  |  |  |
| 1. **I get adequate support when there are problems with the materials.** |  |  |  |  |  |  |
| 1. **I have trust in the quick arrival of the SHS nurse when needed.** |  |  |  |  |  |  |
| 1. **Scheduling appointments with the SHS nurse runs smoothly.** |  |  |  |  |  |  |
| 1. **The SHS nurse is flexible in making appointments.** |  |  |  |  |  |  |
| 1. **I am informed on time when an appointment changes or when a substitute nurse will come.** |  |  |  |  |  |  |
| 1. **I know where to turn to with my questions regarding the SHS.** |  |  |  |  |  |  |
| 1. **In case of problems with the SHS, I can always contact the coordinator.** |  |  |  |  |  |  |

1. **What could be improved regarding the accessibility and communication?**
   - Nothing
   - The following:

|  |
| --- |

1. **Would you like to have an annual evaluation of the home treatment?** (for example, a nurse who visits you once a year to evaluate the home treatment together with you)
   - Yes
   - No
   - I don’t know

**Collaboration**

*The following questions are about the collaboration between the professionals of the Sanquin Home Service and your other health care providers (such as the professionals in the hospital or your general practitioner).*

*Please answer the questions by indicating to which extent you agree with the statements. If you have no experience regarding a particular subject or don’t know the answer, please choose ‘I don’t know / ‘Not applicable’*

|  | ***Strongly disagree*** | ***Partially disagree*** | ***Neither agree, nor disagree*** | ***Partially agree*** | ***Strongly agree*** | ***I don’t know / Not applicable*** |
| --- | --- | --- | --- | --- | --- | --- |
| 1. **My general practitioner is aware of my treatment by the Sanquin Home Service.** |  |  |  |  |  |  |
| 1. **The home treatment matches well with the care I receive from other health care providers.** |  |  |  |  |  |  |
| 1. **I receive contradictory information from health care providers about the Sanquin Home Service.** |  |  |  |  |  |  |

1. **What could be improved regarding the collaboration between health care providers?**
   - Nothing
   - The following:

|  |
| --- |

**Administering medication**

*The following questions concern the administration of the medication at home. For example, injecting or connecting and adjusting the infusion pump.*

*Please answer the questions by indicating to which extent you agree with the statements. If you have no experience regarding a particular subject, please answer ‘Not applicable’*

|  | ***Strongly disagree*** | ***Partially disagree*** | ***Neither agree, nor disagree*** | ***Partially agree*** | ***Strongly agree*** | ***Not applicable*** |
| --- | --- | --- | --- | --- | --- | --- |
| 1. **The administration of the medication runs smoothly.** |  |  |  |  |  |  |
| 1. **I can operate the infusion pump by myself.** |  |  |  |  |  |  |
| 1. **In case of problems regarding the administration of the medication, I ask for help from the nurse of the Sanquin Home Service.** |  |  |  |  |  |  |
| 1. **I know what to do in case of an allergic reaction.** |  |  |  |  |  |  |
| 1. **I sometimes decide to skip an administration.** |  |  |  |  |  |  |
| 1. **I sometimes change the dosage of the medication.** |  |  |  |  |  |  |
| 1. **I feel insecure administering the medication.** |  |  |  |  |  |  |
| 1. **I think the administration mode is unpleasant.** |  |  |  |  |  |  |

1. **Would you say you are easy or difficult to inject?** (For example, because you have small blood vessels.)
   - Very easy
   - Easy
   - Not easy, not difficult
   - Difficult
   - Very difficult
   - I don’t know

**Fear of needles**
*The following questions are about your (the patients’) fear of being injected with needles. When answering these questions, think of the last few times you were injected. A ‘0’ means ‘no fear’ and a ‘10’ refers to ‘worst possible fear’.*

*If you do not have any experience with injections by a particular person, or if you don’t know the answer to the question, please choose "I don’t know / not applicable".*

|  | | | | | | | | | | |  |  |
| --- | --- | --- | --- | --- | --- | --- | --- | --- | --- | --- | --- | --- |
| **How much fear do you experience when injected by…**   1. **…yourself?** | | | | | | | | | | |  |  |
| ***0. No fear*** | ***1.*** | ***2.*** | ***3.*** | ***4.*** | ***5.*** | ***6.*** | ***7.*** | ***8.*** | ***9.*** | ***10. Worst possible fear*** |  | ***I don’t know / Not applicable*** |
|  |  |  |  |  |  |  |  |  |  |  |  |  |
| 1. **… a parent or spouse/ informal caregiver?** | | | | | | | | | | |  |  |
| ***0. No fear*** | ***1.*** | ***2.*** | ***3.*** | ***4.*** | ***5.*** | ***6.*** | ***7.*** | ***8.*** | ***9.*** | ***10. Worst possible fear*** |  | ***I don’t know / Not applicable*** |
|  |  |  |  |  |  |  |  |  |  |  |  |  |
| 1. **… the regular/personal nurse of Sanquin Home Service?** | | | | | | | | | | |  |  |
| ***0. No fear*** | ***1.*** | ***2.*** | ***3.*** | ***4.*** | ***5.*** | ***6.*** | ***7.*** | ***8.*** | ***9.*** | ***10. Worst possible fear*** |  | ***I don’t know / Not applicable*** |
|  |  |  |  |  |  |  |  |  |  |  |  |  |
| 1. **… a substitute nurse of the Sanquin Home Service?** | | | | | | | | | | |  |  |
| ***0. No fear*** | ***1.*** | ***2.*** | ***3.*** | ***4.*** | ***5.*** | ***6.*** | ***7.*** | ***8.*** | ***9.*** | ***10. Worst possible fear*** |  | ***I don’t know / Not applicable*** |
|  |  |  |  |  |  |  |  |  |  |  |  |  |
| 1. **… a nurse in the hospital?** | | | | | | | | | | |  |  |
| ***0. No fear*** | ***1.*** | ***2.*** | ***3.*** | ***4.*** | ***5.*** | ***6.*** | ***7.*** | ***8.*** | ***9.*** | ***10. Worst possible fear*** |  | ***I don’t know / Not applicable*** |
|  |  |  |  |  |  |  |  |  |  |  |  |  |

**Benefits of the Sanquin Home Service***What are benefits of the Sanquin Home Service, as compared to treatment in the hospital?*

*Please answer the questions by indicating to which extent you agree with the statements. If you have no experience regarding a particular subject or if you don’t know the answer, please choose ‘I don’t know / ‘Not applicable’.*

| 1. **I think it’s a benefit of the Sanquin Home Service (compared to hospital based treatment) that…** | ***Strongly disagree*** | ***Partially disagree*** | ***Neither agree, nor disagree*** | ***Partially agree*** | ***Strongly agree*** | ***I don’t know / Not applicable*** |
| --- | --- | --- | --- | --- | --- | --- |
| 1. **…I can be treated in my own home environment.** |  |  |  |  |  |  |
| 1. **…I don’t have to be in the hospital.** |  |  |  |  |  |  |
| 1. **…I don’t have to travel.** |  |  |  |  |  |  |
| 1. **…I am less vulnerable to hospital acquired infections.** |  |  |  |  |  |  |
| 1. **…I can choose the time and day of the infusion.** |  |  |  |  |  |  |
| 1. **…the product is administered at a proper temperature.** |  |  |  |  |  |  |
| 1. **…the infusion rate of the medication is adapted to my preference.** |  |  |  |  |  |  |
| 1. **…the nurses of the Sanquin Home Service have good knowledge about the medication.** |  |  |  |  |  |  |
| 1. **…I know the nurses of the Sanquin Home Service well.** |  |  |  |  |  |  |

1. **Do you experience other benefits of the Sanquin Home Service?**
   - No
   - Yes:

|  |
| --- |

**Health and quality of life**

*What impact does the Sanquin Home Service have on your health and quality of life, in comparison with treatment at the hospital?*

*.*

*Please answer the following questions by indicating to which extent you agree with the statements. If you have no experience regarding a particular subject, or if you don’t know the answer or don’t want to answer the question, please choose ‘I don’t know / ‘No answer’.*

| 1. **Because of the home treatment …** | ***Strongly disagree*** | ***Partially disagree*** | ***Neither agree, nor disagree*** | ***Partially agree*** | ***Strongly agree*** | ***I don’t know / Not applicable*** | |
| --- | --- | --- | --- | --- | --- | --- | --- |
| 1. **… I live a more normal life.** |  |  |  |  |  |  |  |
| 1. **… I feel less like a patient.** |  |  |  |  |  |  |  |
| 1. **… I feel less dependent of health care providers.** |  |  |  |  |  |  |  |
| 1. **… I feel more free.** |  |  |  |  |  |  |  |
| 1. **… I can live the life I want.** |  |  |  |  |  |  |  |
| 1. **… my daily life is less disrupted by my disease.** |  |  |  |  |  |  |  |
| 1. **… I have less trouble with work or school.** |  |  |  |  |  |  |  |
| 1. **… I feel less sick.** |  |  |  |  |  |  |  |
| 1. **… I feel safer.** |  |  |  |  |  |  |  |
| 1. **… my health is more stable.** |  |  |  |  |  |  |  |
| 1. **… I have less physical problems.** |  |  |  |  |  |  |  |
| 1. **… my body is less burdened.** |  |  |  |  |  |  |  |
| 1. **… I have more energy.** |  |  |  |  |  |  |  |
| 1. **… I have a better quality of life.** |  |  |  |  |  |  |  |

1. **How are you doing since the start of the Sanquin Home Service?
   Because of the Sanquin Home Service, as compared to hospital based treatment, I am doing:**
   - Much better
   - Better
   - Somewhat better
   - The same
   - Somewhat worse
   - Worse
   - Much worse
   - I don’t know

**Overall ratings of the Sanquin Home Service and hospital care**

*What is your general opinion on the Sanquin Home Service and the treatment at the hospital?*

1. **How do you rate the Sanquin Home Service? (0=very bad care; 10=excellent care)**
   - 0 Very bad care
   - 1
   - 2
   - 3
   - 4
   - 5
   - 6
   - 7
   - 8
   - 9
   - 10 Excellent care
2. **Would you recommend the Sanquin Home Service to other patients? (0=definitely not; 10=definitely yes)**
   - 0 Definitely not
   - 1
   - 2
   - 3
   - 4
   - 5
   - 6
   - 7
   - 8
   - 9
   - 10 Definitely yes
3. **How do you rate the hospital care? (0=very bad care; 10=excellent care)**
   - 0 Very bad care
   - 1
   - 2
   - 3
   - 4
   - 5
   - 6
   - 7
   - 8
   - 9
   - 10 Excellent care
   - Not applicable, I never received care in the hospital

**Patient characteristics**

*The following questions concern the background characteristics of the patient.*

1. **Gender:**
   - Male
   - Female
2. **Age:**

____ years

1. **Educational level:**

- No education
- Elementary education
- Pre-vocational education
- Intermediate general education
- Intermediate vocational education
- Selective secondary education
- High vocational education
- Academic education
- Other:

1. **How is your general health? (health of the patient)**
   - Excellent
   - Very good
   - Good
   - Moderate
   - Poor
2. **Did someone help to complete the questionnaire?**
   - No (completed by the patient)
   - Yes, spouse
   - Yes, parent
   - Yes, someone else: …………………………………………………….

**Questions for parents and informal caregivers**

*These final questions are meant for parents and informal caregivers (family member, spouse or someone else who assists in the home treatment).*

1. **Are you a parent or an informal caregiver?**
   - Parent
   - Informal caregiver

*The following questions concern your experiences with the Sanquin Home Service and the support you receive as a parent or informal caregiver. For example, in the guidance of the treatment or in case of problems with injecting the medication.*

1. **What is your role in the administration of medication?** *(multiple answers possible)*
   - I help with the preparation of the infusion (e.g. filling, disinfecting)
   - I inject the medication
   - I am present (at home) when the medication is being administered
   - I assist at the end, with disconnecting and disinfecting
   - I do not contribute at all (the SHS nurse does everything) *[-> question 104]*

*Please answer the questions by indicating to which extent you agree with the statements. If you have no experience regarding a particular subject, answer ‘I don’t know / ‘Not applicable’.*

| 1. **The nurse of the Sanquin Home Service…** | ***Strongly disagree*** | ***Partially disagree*** | ***Neither agree, nor disagree*** | ***Partially agree*** | ***Strongly agree*** | ***I don’t know / Not applicable*** |
| --- | --- | --- | --- | --- | --- | --- |
| 1. **…guides me well in the treatment of the patient.** |  |  |  |  |  |  |
| 1. **…advises me well in case of problems with the treatment.** |  |  |  |  |  |  |
| 1. **I was closely involved in the decision whether or not to use the Sanquin Home Service** |  |  |  |  |  |  |
| 1. **I feel free to choose whether or not the help with administering the medication** |  |  |  |  |  |  |

| **Questions for parents:**   1. **The nurse of the Sanquin Home Service ….** | ***Strongly disagree*** | ***Partially disagree*** | ***Neither agree, nor disagree*** | ***Partially agree*** | ***Strongly agree*** | ***I don’t know / Not applicable*** |
| --- | --- | --- | --- | --- | --- | --- |
| 1. **…could manage my child well.** |  |  |  |  |  |  |
| 1. **…advises me well if there are any problems with the treatment of my child.** |  |  |  |  |  |  |
| 1. **…teaches me, as a parent, how to deal with problems regarding injecting the medication.** |  |  |  |  |  |  |
| 1. **…guides me well in being able to administer the medication to my child.** |  |  |  |  |  |  |
| 1. **…assists or teaches my child in administering the medication independently (by him-/herself).** |  |  |  |  |  |  |

**Thank you for completing this questionnaire!**
