# Supplementary material for: Home-Based Treatment with Immunoglobulins: an Evaluation from the Perspective of Patients and Healthcare Professionals
Source: J Clin Immunol. 2018 Nov 12;38(8):876–85. doi: 10.1007/s10875-018-0566-z (PMC6292972; doi:10.1007/s10875-018-0566-z)
Supplement: Supplementary file 2 — (DOCX 108 kb) [file 10875_2018_566_MOESM2_ESM.docx]

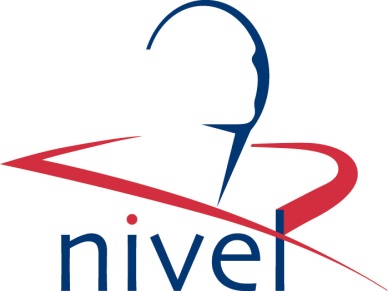
 **Appendix 2.**


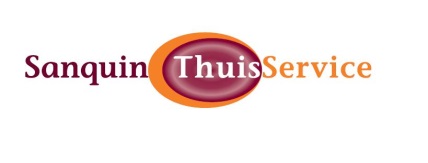


Questionnaire

**Sanquin Home Service**

Experiences of professionals

**Note:**

This is a translation of a Dutch digital questionnaire for nurses of the Sanquin Home Service and hospital professionals. The original questionnaire is included as an appendix in the Dutch research report (Triemstra et al., 2015) which can be downloaded from the Nivel-website (www.nivel.nl).

**General questions**

1. **What is your occupation?**
   - Nurse of the Sanquin Home Service *[-> question 3]*
   - Physician at a hospital
   - Nurse at a hospital
   - Other health care professionals at the hospital: …………
   - Other: ………………………………………………………………….
2. **At which hospital department do you work?**
   - Neurology
   - Immunology
   - Hematology
   - Infectious diseases
   - Other: ………………………………………………………………….
3. **Are you a man or a woman?**
   - Man
   - Woman
4. **What is your age?**

____ years

**Involvement in the Sanquin Home Service**

*The following questions are about your involvement in the Sanquin Home Service. Either as a professional (doctor / nurse) at the hospital, or as a nurse working for the home service.*

1. **How long have you been professionally involved with the Sanquin Home Service?**
   - Less than a year
   - 1 to 3 years
   - 3 to 6 years
   - 6 to 10 years
2. **How many of your patients currently receive Sanquin Home Service?** *This concerns patients who receive immunoglobulins at home. Please estimate.*

… patients

1. **How often do you have contact by phone or e-mail with the coordinators of the Sanquin Home Service?**
   - Several times a week
   - Once a week (weekly)
   - Once every two weeks (biweekly)
   - Once every three weeks
   - Once a month (monthly)
   - A few times a year
   - Irregularly
   - Never

**Experiences with the Sanquin Home Service***The following questions are about your own experiences, as a professional, with the Sanquin Home Service.*

**Questions for SHS nurses**

1. **Contact with patients**

*These questions are about your contact with patients. Please answer them by indicating to which extent you agree with the statements. If you have no experience regarding a particular subject or if you don’t know the answer, please choose ‘I don’t know / not applicable’.*

|  | ***Strongly disagree*** | ***Partially disagree*** | ***Neither agree, nor disagree*** | ***Partially agree*** | ***Strongly agree*** | ***I don’t know / Not applicable*** |
| --- | --- | --- | --- | --- | --- | --- |
| 1. **I get along well with the patients I visit at home (it’s a perfect match).** |  |  |  |  |  |  |
| 1. **I like being able to build a personal relation with patients.** |  |  |  |  |  |  |
| 1. **I sometimes think I adjust to much to my patients.** |  |  |  |  |  |  |
| 1. **I inject my patients well.** |  |  |  |  |  |  |
| 1. **I have sufficient time for my patients.** |  |  |  |  |  |  |
| 1. **I know what to do in case of emergencies (e.g. allergic reactions).** |  |  |  |  |  |  |
| 1. **I can quickly arrive at the patient’s home when needed.** |  |  |  |  |  |  |
| 1. **There are sufficient practice sessions before patients start to administer the medication themselves (subcutaneously).** |  |  |  |  |  |  |
| 1. **I can properly guide children to administer the medication independently (subcutaneously).** |  |  |  |  |  |  |
| 1. **I can guide parents well in the home treatment of their child.** |  |  |  |  |  |  |
| 1. **I can mediate conflicts between patients and informal caregivers (parents of spouses) with regard to the home treatment.** |  |  |  |  |  |  |

1. **What could be improved in the contact with patients?**
   - Nothing
   - The following:

|  |
| --- |

1. **Appointments with patients**

*These questions concern your appointments with patients. Please answer them by indicating to which extent you agree with the statements. If you have no experience regarding a particular subject or don’t know the answer, please choose ‘I don’t know / not applicable’.*

|  | ***Strongly disagree*** | ***Partially disagree*** | ***Neither agree, nor disagree*** | ***Partially agree*** | ***Strongly agree*** | ***I don’t know / Not applicable*** |
| --- | --- | --- | --- | --- | --- | --- |
| 1. **Scheduling appointments with my patients runs smoothly.** |  |  |  |  |  |  |
| 1. **I am flexible in making appointments.** |  |  |  |  |  |  |
| 1. **I visit my patients exclusively during office hours (9 am to 5 pm).** |  |  |  |  |  |  |
| 1. **Patients know that appointments for home treatment preferably take place during office hours.** |  |  |  |  |  |  |
| 1. **Occasionally, I visit my patients after office hours when needed.** |  |  |  |  |  |  |

1. **What could be improved regarding the appointments with patients?**
   - Nothing
   - The following:

|  |
| --- |

**Communication and collaboration***The following questions concern the communication and collaboration with other health care providers.*

*Please answer the questions by indicating to which extent you agree with the statements. If you have no experience regarding a particular subject or if you don’t know the answer, please choose ‘I don’t know / not applicable’.*

**Questions for all professionals**

|  | ***Strongly disagree*** | ***Partially disagree*** | ***Neither agree, nor disagree*** | ***Partially agree*** | ***Strongly agree*** | ***I don’t know / Not applicable*** |
| --- | --- | --- | --- | --- | --- | --- |
| 1. **Patients receive sufficient information from hospital professionals about the Sanquin Home Service.** |  |  |  |  |  |  |
| 1. **Patients know well what to expect from the home treatment before the start of Sanquin Home Service.** |  |  |  |  |  |  |
| 1. **There is a smooth transition from treatment in the hospital to treatment at home.** |  |  |  |  |  |  |
| 1. **The patients' medical records are handed over well in the transition from hospital-based treatment to treatment at home.** |  |  |  |  |  |  |
| 1. **Patients get all the information they need from the coordinators of Sanquin Home Service.** |  |  |  |  |  |  |
| 1. **Patients are well informed about the pharmacy’s delivery at home.** |  |  |  |  |  |  |
| 1. **I am well informed by the SHS coordinators about relevant matters concerning the home treatment.** |  |  |  |  |  |  |
| 1. **I am well aware of protocols and procedures of the Sanquin Home Service.** |  |  |  |  |  |  |
| 1. **I can answer all of the patients’ questions about the organization of the Sanquin Home Service.** |  |  |  |  |  |  |
| 1. **I can answer all of the patients’ questions about the treatment by the Sanquin Home Service.** |  |  |  |  |  |  |
| 1. **Patients know where to turn to with questions regarding the SHS.** |  |  |  |  |  |  |

*The nurses who work for the Sanquin Home Service are referred to as 'SHS nurses' hereafter.*

*Please answer the questions by indicating to which extent you agree with the statements. If you have no experience regarding a particular subject or if you don’t know the answer, please choose ‘I don’t know / not applicable’.*

**Questions for SHS-nurses**

|  | ***Strongly disagree*** | ***Partially disagree*** | ***Neither agree, nor disagree*** | ***Partially agree*** | ***Strongly agree*** | ***I don’t know / Not applicable*** |
| --- | --- | --- | --- | --- | --- | --- |
| 1. **Me and other SHS nurses collaborate well.** |  |  |  |  |  |  |
| 1. **In case of absence due to illness or holidays, my patients are well cared for by a substitute nurse.** |  |  |  |  |  |  |
| 1. **I have direct communication lines with the coordinators of the Sanquin Home Service.** |  |  |  |  |  |  |
| 1. **I have direct communication lines with the hospital professionals.** |  |  |  |  |  |  |
| 1. **I am well informed by the SHS about relevant matters concerning my patients (e.g. check-ups, lab tests, hospitalizations).** |  |  |  |  |  |  |

**Questions for all professionals**

1. **What could be improved regarding the communication and collaboration?**
   - Nothing
   - The following:

|  |
| --- |

**Reporting and information exchange**

*The following questions concern the exchange of information with other health care providers.*

*Please answer the questions by indicating to which extent you agree with the statements. If you have no experience regarding a particular subject or if you don’t know the answer, please choose ‘I don’t know / not applicable’.*

|  | ***Strongly disagree*** | ***Partially disagree*** | ***Neither agree, nor disagree*** | ***Partially agree*** | ***Strongly agree*** | ***I don’t know / Not applicable*** |
| --- | --- | --- | --- | --- | --- | --- |
| 1. **I can easily find information regarding my patients in MijnThuisService.nl.** |  |  |  |  |  |  |
| 1. **MijnThuisService.nl is a good system for exchanging information between me and the coordinators of the Sanquin Home Service.** |  |  |  |  |  |  |

**Questions for SHS-nurses**

|  | ***Strongly disagree*** | ***Partially disagree*** | ***Neither agree, nor disagree*** | ***Partially agree*** | ***Strongly agree*** | ***I don’t know / Not applicable*** |
| --- | --- | --- | --- | --- | --- | --- |
| 1. **It is clear to me how to report to the Sanquin Home Service.** |  |  |  |  |  |  |
| 1. **I report at least twice a year to the Sanquin Home Service.** |  |  |  |  |  |  |
| 1. **As a substitute nurse, I am well informed about the patients.** |  |  |  |  |  |  |
| 1. **I am well aware of the personal situation of my patients.** |  |  |  |  |  |  |

**Question for all professionals**

1. **What could be improved regarding the information exchange?**
   - Nothing
   - The following:

|  |
| --- |

**Benefits of the Sanquin Home Service**

*What are benefits of the Sanquin Home Service, compared to treatment in the hospital?*

*Please answer the following questions by indicating to which extent you agree with the statements. If you have no experience regarding a particular subject or if you don’t know the answer, please choose ‘I don’t know / not applicable’.*

| 1. **I think it’s a benefit of the Sanquin Home Service (compared to hospital based treatment) that…** | ***Strongly disagree*** | ***Partially disagree*** | ***Neither agree, nor disagree*** | ***Partially agree*** | ***Strongly agree*** | ***I don’t know / Not applicable*** |
| --- | --- | --- | --- | --- | --- | --- |
| 1. **… patients receive treatment in their own home environment.** |  |  |  |  |  |  |
| 1. **… patients don’t have to travel to the hospital for treatment.** |  |  |  |  |  |  |
| 1. **… patients are less vulnerable to hospital acquired infections.** |  |  |  |  |  |  |
| 1. **… patients spend less time on the treatment.** |  |  |  |  |  |  |
| 1. **… patients can choose the time and day of the infusion.** |  |  |  |  |  |  |
| 1. **… administration of the medication is possible outside office hours.** |  |  |  |  |  |  |
| 1. **… patients can adapt the temperature of the product (to room temperature at the time of administration).** |  |  |  |  |  |  |
| 1. **… the SHS nurses are competent in administering injections.** |  |  |  |  |  |  |
| 1. **… the SHS nurses can take sufficient time for patients.** |  |  |  |  |  |  |
| 1. **… the SHS nurses can adapt to the needs of patients.** |  |  |  |  |  |  |
| 1. **… patients know the SHS nurses well.** |  |  |  |  |  |  |
| 1. **… patients have a personal SHS nurse.** |  |  |  |  |  |  |
| 1. **… the SHS nurses have good knowledge about the medication.** |  |  |  |  |  |  |
| 1. **… the SHS nurses know how to administer the medication.** |  |  |  |  |  |  |

1. **Do you experience other benefits of the Sanquin Home Service?**
   - No
   - Yes:

|  |
| --- |

**Effects of the Sanquin Home Service on patients**

*The following questions concern the impact of the Sanquin Home Service on the patients’ life. What are the effects of the home treatment on patients, according to you?*

*Please answer the questions by indicating to which extent you agree with the statements. If you have no experience regarding a particular subject or if you don’t know the answer, please choose ‘I don’t know / not applicable’.*

| 1. **Because of the Sanquin Home Service, patients…** | ***Strongly disagree*** | ***Partially disagree*** | ***Neither agree, nor disagree*** | ***Partially agree*** | ***Strongly agree*** | ***I don’t know / Not applicable*** |
| --- | --- | --- | --- | --- | --- | --- |
| 1. **… feel less like a patient.** |  |  |  |  |  |  |
| 1. **… can better fit the treatment in their daily life.** |  |  |  |  |  |  |
| 1. **.. have more control over their life.** |  |  |  |  |  |  |
| 1. **… are more compliant to using their medication.** |  |  |  |  |  |  |
| 1. **… experience less adverse effects.** |  |  |  |  |  |  |
| 1. **… have more energy.** |  |  |  |  |  |  |
| 1. **… experience less burden to their body.** |  |  |  |  |  |  |
| 1. **… have less trouble with school or work.** |  |  |  |  |  |  |
| 1. **… have a more stable health.** |  |  |  |  |  |  |
| 1. **… have less physical problems.** |  |  |  |  |  |  |
| 1. **… have a better quality of life.** |  |  |  |  |  |  |

**Working with patients from the Sanquin Home Service**

*The following questions are about working with patients from the Sanquin Home Service.*

*Please answer the questions by indicating to which extent you agree with the statements. If you have no experience regarding a particular subject or if you don’t know the answer, please choose ‘I don’t know / not applicable’.*

**Questions for SHS nurses**

|  | ***Strongly disagree*** | ***Partially disagree*** | ***Neither agree, nor disagree*** | ***Partially agree*** | ***Strongly agree*** | ***I don’t know / Not applicable*** |
| --- | --- | --- | --- | --- | --- | --- |
| 1. **I like working with patients from the Sanquin Home Service.** |  |  |  |  |  |  |
| 1. **I receive appreciation for my work from patients.** |  |  |  |  |  |  |
| 1. **I receive appreciation for my work form the Sanquin Home Service.** |  |  |  |  |  |  |
| 1. **I receive appreciation for my work from colleagues at the hospital.** |  |  |  |  |  |  |
| 1. **I will persevere my work for patients from the Sanquin Home Service until my retirement.** |  |  |  |  |  |  |

|  | ***Strongly disagree*** | ***Partially disagree*** | ***Neither agree, nor disagree*** | ***Partially agree*** | ***Strongly agree*** | ***I don’t know / Not applicable*** |
| --- | --- | --- | --- | --- | --- | --- |
| 1. **I am proud to work for the Sanquin Home Service.** |  |  |  |  |  |  |
| 1. **I feel involved with the Sanquin Home Service.** |  |  |  |  |  |  |
| 1. **The Sanquin ThuisService listens to what I find important.** |  |  |  |  |  |  |
| 1. **The Sanquin Home Service takes my wishes and needs as a professional into account.** |  |  |  |  |  |  |

**Question for all professionals**

*Please answer the questions by indicating to which extent you agree with the statements. If you have no experience regarding a particular subject or if you don’t know the answer, please choose ‘I don’t know / not applicable’.*

|  | ***Strongly disagree*** | ***Partially disagree*** | ***Neither agree, nor disagree*** | ***Partially agree*** | ***Strongly agree*** | ***I don’t know / Not applicable*** |
| --- | --- | --- | --- | --- | --- | --- |
| 1. **The Sanquin Home Service has enriched and broadened my work.** |  |  |  |  |  |  |
| 1. **I think that home treatment like the Sanquin Home Service is cheaper than treatment in the hospital.** |  |  |  |  |  |  |
| 1. **I expect a growing demand for home treatment with immunoglobulins in the future.** |  |  |  |  |  |  |
| 1. **The Sanquin Home Service has become indispensable.** |  |  |  |  |  |  |

**Overall rating of the Sanquin Home Service**

*The following questions are about your general opinion on the Sanquin Home Service.*

1. **How do you rate the Sanquin Home Service? (0=very bad care; 10 excellent care)**
   - 0 Very bad care
   - 1
   - 2
   - 3
   - 4
   - 5
   - 6
   - 7
   - 8
   - 9
   - 10 Excellent care
2. **Would you recommend the Sanquin Home Service to other patients? (0=definitely not; 10=definitely yes)**
   - 0 Definitely not
   - 1
   - 2
   - 3
   - 4
   - 5
   - 6
   - 7
   - 8
   - 9
   - 10 Definitely

**Question for hospital professionals**

1. **I choose the Sanquin Home Service because ...**

|  |
| --- |

- - No answer

**Thank you for completing this questionnaire!**
