# Supplementary material for: Home-Based Treatment with Immunoglobulins: an Evaluation from the Perspective of Patients and Healthcare Professionals
Source: J Clin Immunol. 2018 Nov 12;38(8):876–85. doi: 10.1007/s10875-018-0566-z (PMC6292972; doi:10.1007/s10875-018-0566-z)
Supplement: Supplementary file 3 — (DOCX 25 kb) [file 10875_2018_566_MOESM3_ESM.docx]

**Appendix 3.** Overview of scales and corresponding items in the questionnaires for clients and professionals (with Cronbach’s alpha of each scale)

| Clients’ questionnaire: Experiences | | | | | | | | |
| --- | --- | --- | --- | --- | --- | --- | --- | --- |
| *Start of SHS: transition and information (alpha = .78)*   - The transition from hospital treatment to home treatment went well. - Beforehand, I knew what to expect from the home treatment.   Beforehand, I got sufficient information regarding...   - the processes of SHS. - delivery of medication and materials at home. - the home treatment’s costs. | | ***Start of SHS: practice sessions*** ***and self-infusion*** *(alpha = .70)*   - After the final practice session, I was confident to infuse myself. - During the starting period, I was uncomfortable infusing myself. - During the starting period, I thought the self-infusion was scary. | | | ***Contact with SHS-coordinators*** *(alpha = .90)*  The coordinator of the SHS…:   - takes sufficient time for me. - is aware of my situation. - listens to me carefully. - takes me seriously. - takes my wishes and needs into account. - answers my questions well. - I have faith in the expertise of the coordinator. - I feel emotionally supported by the coordinator. | | | ***Contact with SHS-nurses*** *(alpha =.93)*  The SHS nurse….:   - takes sufficient time for me. - is aware of my situation. - listens to me carefully. - takes me seriously. - takes my wishes and needs into account. - could answer all my questions about the home treatment. - is competent in administering injections. - works hygienically. - I have faith in the expertise of the nurse. - I feel emotionally supported by the nurse. |
| *Distribution of medication (alpha = .88)*   - The delivery of medication runs smoothly. - I always know exactly what type and dosage of medication will be delivered. - Scheduling appointments for the delivery of medication runs smoothly. - I am well informed about the time of delivery. - Medication is delivered in the agreed time period. - I am informed on time if the medication cannot be delivered in the agreed time period. - Medication is delivered on time. - Dosages are always delivered correctly. - I know what to do when there are problems with the medication delivery. | | ***Distribution of materials*** *(alpha = .92)*   - The delivery of materials runs smoothly. - Scheduling appointments for the delivery of materials runs smoothly. - Materials are delivered on time. - I am well informed about the time of delivery. - Materials are delivered in the agreed time period. - I am informed on time if materials cannot be delivered in the agreed time period. - I have enough space to store all of the materials at home. - I know what to do when there are problems with the delivery of materials. | | | ***Accessibility*** ***and communication*** *(alpha = .82)*   - The SHS coordinators are available when needed. - Telephone contact with the coordinators is running smoothly. - The SHS nurse is easily accessible. - Telephone contact with the SHS nurse is running smoothly. - I get adequate support when there are problems with the medication. - I get adequate support when there are problems with the materials. - I have trust in the quick arrival of the SHS nurse when needed. - Scheduling appointments with the SHS nurse runs smoothly. - I know where to turn to with my questions regarding the SHS. - The SHS nurse is flexible in making appointments. - I am informed on time when an appointment changes or when a substitute nurse will come. - In case of problems with the SHS, I can always contact the coordinator. | | | |
| Professionals’ questionnaire Experiences | | | | | | | | |
| *Start of SHS: transition and information to patients (alpha = .84)*   - Patients receive sufficient information about the SHS from hospital professionals. - Patients know well what to expect from the home treatment before the start of SHS. - There is a smooth transition from treatment in the hospital to treatment at home. - The patients’ medical records are handed over well in the transition from hospital to home treatment. - Patients get all the information they need from the SHS coordinators. - Patients are well informed about the pharmacy’s delivery at home. - Patients know where to turn to with their questions regarding the SHS. | | ***Knowledge of SHS*** *(alpha = .81)*   - I am well informed by the SHS coordinators about relevant matters concerning the home treatment. - I am well aware of protocols and procedures of the SHS. - I can answer all of the patients’ questions about the SHS organization. - I can answer all of the patients’ questions about the home treatment. | | |  |  | | |
| Clients’ questionnaire: Benefits and Effects | | | | | | | | |
| *Benefit: No hospitalization (alpha = .72)*  I think it’s a benefit of SHS (as opposed to hospital treatment) that…:   - I can be treated in my own home environment. - I don’t have to be in the hospital. - I don’t have to travel. - I am less vulnerable to hospital acquired infections. | ***Benefit: Greater sense of self control*** *(alpha = .76)*  I think it’s a benefit of SHS (as opposed to hospital treatment) that…:   - I can choose the time and day of infusion. - the product is administered at a proper temperature. - the infusion rate of the medication is adapted to my preference. | | | ***Effect: Health and physical functioning*** *(alpha = .94)*  Because of the treatment at home (as opposed to hospital treatment)…:   - I live a more normal life. - I feel less like a patient. - I feel less dependent on healthcare providers. - I feel more free. - I can live the life I want. - my daily life is less disrupted by my disease. | | | ***Effect: Autonomy and participation*** *(alpha = .90)*  Because of the treatment at home (as opposed to hospital treatment)…:   - I feel less sick. - I feel safer. - my health is more stable. - I have less physical problems. - my body is less burdened. - I have more energy. | |
| Professionals’ questionnaire: Benefits and Effects | | | | | | | | |
| *Benefit: High quality nursing (alpha = .92)*  It’s a benefit of the SHS that…:   - the SHS nurses are competent in administering injections. - the SHS nurses can take sufficient time for patients. - the SHS nurses can adapt to the needs of patients. - patients know the SHS nurses well. - patients have a personal SHS nurse. - the SHS nurses have good knowledge about the medication. - the SHS nurses know how to administer the medication. | | | | ***Effect: Quality of life*** *(alpha = .94)*  Because of the SHS, patients…:   - feel less like a patient. - can fit the treatment in their daily life. - have more control over their life. - are more compliant to using their medication. - experience less adverse effects. - have more energy. - experience less burden to their body. - have less trouble with school or work. - have a more stable health. - have less physical problems. - have a better quality of life. | | | | |
| Both questionnaires: scales with similar items (for comparing the perspectives of clients and professionals) | | | | | | | | |
| *Transition and information provided (alpha = .73)*   - Smooth transition from hospital to home treatment. - Knowing what to expect before the start of the SHS home treatment. - Patients get sufficient information from the hospital professionals. - Patients get sufficient information from the SHS coordinators. | ***Contact between patients and SHS nurses*** *(alpha =.73)*   - Getting along well. - Sufficient time. - The SHS nurse is competent in administering injections. - The SHS nurse answers all the questions regarding home treatment well. | | ***Accessibility and communication*** *(alpha =.61)*   - Patients know where to turn to with their questions. - Quick arrival at the patients home when needed. - Scheduling appointments runs smoothly. - The SHS nurse is flexible in making appointments. | | |  | | |
| *Perceived benefits of SHS* (alpha clients = .73 / professionals = .58)  Compared to hospital treatment, I consider it a benefit of SHS home treatment that…:   - the patient is treated at home. - the patient does not have to be in the hospital. - the patient is less susceptible to hospital infections. - the patient can choose the day and time of infusion. - the product is administered at a proper temperature. - patients know the SHS nurse well. - SHS nurses have good knowledge about the medication. | | | ***Perceived effect of SHS on quality of life*** *(alpha clients = .92 / professionals = .95)*  Because of the treatment at home (as opposed to hospital treatment) patients…:   - feel less like a patient. - have less trouble with school or work. - have a more stable health. - have less physical problems. - experience less burden to their body. - have more energy. - have a better quality of life. | | | | | |
